# Different impact on health outcomes of long-term care insurance between urban and rural older residents in China

**DOI:** 10.1038/s41598-023-27576-6

**Published:** 2023-01-05

**Authors:** Paicheng Liu, Youlang Yang, Yuxuan Yang, Jianxin Cheng

**Affiliations:** 1 School of Public Administration, Southwestern University of Finance and Economics, Chengdu, China; 2 School of Government, Sun Yat-Sen University, Guangzhou, China; 3 School of Public Administration and Emergency Management, Jinan University, Guangzhou, China

**Keywords:** Geriatrics, Health policy

## Abstract

Long-term care insurance (LTCI) is garnering attention internationally and is being considered a public policy in a growing number of countries. Previous research has focused on the effects of LTCI in developed countries, ignoring the health outcomes of developing countries, especially in rural regions. Therefore, this study investigates whether different impact on health outcomes is present in the effects of LTCI between urban and rural residents in China. We employed a quasi-experimental design with data from the China Health and Retirement Longitudinal Survey. The specific implementation time of each pilot city was sorted according to the LTCI policy texts, dividing these pilot cities into the treatment group and control group. Finally, difference-in-differences analyses were utilized to evaluate the health effects of LTCI between urban and rural residents, and the health effect in urban areas was further tested. The implementation of LTCI has effectively enhanced the self-rating health (SRH) of the entire group of residents; however, this effect may only be significant for the urban group. In particular, LTCI can increase the SRH of urban residents by 0.377 units compared to the urban residents without LTCI (*P* < 0.01). The result of the placebo effect test further verifies that LTCI could improve the health of residents to some extent. In China, LTCI may have triggered different impacts on health outcomes between urban and rural residents, and may not improve the SRH of rural residents and only prove efficacious for urban residents. Government and policy-makers should give more attention to the rural group as it needs long-term care the most.

## Introduction

### LTCI in China

According to the Seventh National Census in China, the number of older people over 65 years of age is approximately 190 million, accounting for 13.5% of the total population^1^. In 2020, the number of older adults with disabilities reached 52.71 million^2^. Given the enlarging demand for long-term care (LTC) for disabled elderly people^3^, the central government of China has initiated a long-term care insurance (LTCI) policy in 29 pilot cities since 2016 to advance the health and well-being of the aforementioned groups and provide them with high-quality integrated medical care. With the deepening aging of the society, it is crucial to increase formal services for the oldest old^4^.

Compared to developed countries, such as Germany, Japan, and South Korea^5–7^, China’s LTCI was launched relatively late and primarily provides fundamental life care services^8^. The leading aim of LTCI in China is to emulate Germany covering all public medical insurance recipients regardless of disabilities and age^9^. Elderly care in Chinese society has been regarded as the family’s responsibility instead of a social problem, however, after the turn of the millennium, the one-child policy and drastic changes in traditional family structure engendered a switch from informal family-based elderly care to the national formal LTC^10^. On the one hand, formal LTC services have increased to meet the escalating demand for older adults^11^. On the other hand, LTC devotes to improving the health and quality of life for older adults, especially for the disabled population, which further supplements family care^12^.

### Benefits and shortcomings of LTCI

Scholars have a growing awareness that LTCI delivers many benefits for society, caregivers, and older adults in general. The outcomes of the LTCI policy reveal that the burden on families caring for older adults has been reduced through social solidarity^13^. The demand for formal care is intensifying due to an eroding traditional Chinese family support system in tandem with the prevalence of a smaller-sized family structure. Accordingly, LTCI could effectively replace informal care, saving time for children^12^, benefit all members of the household, along with both care recipient and caregivers^14^, and produce a positive spillover effect on the labor participation of family caregivers^15^. More importantly, the introduction of LTCI has remarkably reduced the outpatient visits, length of stay, and expenses of older patients in hospitals^16–18^. Notably, disabled older adults enjoy a superior quality of life because LTCI improved their self-rated health (SRH) and mental health^19,20^. For the groups of paramedics and social workers, more employment opportunities have emerged accompanying LTCI^13^.

The formal care services provided by LTCI should target complementing informal care in the short run and decrease inequality in social care in the long run^21^. However, previous research outlines that LTCI has many shortcomings in terms of health equality; for example, LTCI may not be advantageous for the group who truly need long-term care^22^. The current coverage of Chinese elderly care services via LTCI may still be very limited^23^, and financial support for low-income groups is insufficient as well^24^. Although more than 72% of respondents feel satisfied with LTCI, there are disparities between China’s eastern and western regions^25^. Rural and indigent older adults obtained less welfare compared to urban older adults^26^, which might be related to the considerable discrepancy in the financial burden among individuals^27^. The inefficient rural healthcare service provision system and lack of sufficient financing have challenged the old-age health security in rural China^28^.

### Financing mechanism of LTCI in China

China adopts a hybrid scheme for LTCI financing, distinct from Japan and Germany’s social insurance principles to implement mandatory LTCI^21,29^. All the LTCI schemes contain the insured of Urban Employee Basic Medical Insurance (UEBMI) and only a limited proportion additionally contain the insured of Urban Resident Basic Medical Insurance (URBMI) and New Rural Cooperative Medical Scheme (for rural residents)^30^. Institutional LTC services under the existing arrangement are predominantly publicly financed as well as privately funded places, with the financing model of Qingdao City being the most desirable policy option in China^23^. However, a large number of elders in need of care have been excluded due to the specific access barriers arising from the hukou system, which might be more apparent in rural areas^31^. Consequently, some scholars have criticized that the central government should bear certain fiscal responsibility by conducting financial transfers to partially support rural regions^32^. Government subsidies can play a role in stimulating the utilization of formal LTC^33^. Whether the financing of LTCI in China has triggered different impacts on health outcomes between urban and rural elderly residents is thought-provoking.

### Aims of this study

To sum up, extant studies on LTCI in China have accumulated considerable achievements. Yet, little is known about the health outcomes of beneficiaries, especially the difference of which between urban and rural older adults. Therefore, we employed the difference-in-differences (DID) method, covering 12 pilot cities in the first batch to investigate the discrepant impact of LTCI on health. This study contributes to the existing literature in three aspects. Firstly, previous research associated with LTCI generally concentrated on a single city or a limited period. LTCI heretofore has been implemented in China for approximately six years, involving 27 provincial administrative units. Despite multiple benefits of LTCI having been testified by scholars, there is an urgent call for more attention to different impact between urban and rural older adults. The ultimate goal of LTCI is to improve the health and quality of life for older adults. Regrettably, analysis of the health outcomes of LTCI among rural sample is currently scarce. Therefore, we insist that it is necessary to certify whether this policy virtually produces positive health outcomes for rural older adults.

Secondly, medical insurance has attested to provoke higher health inequality for the rural populations^34^. As a part of medical insurance, has the LTCI likewise generated disparate impacts on health outcomes between urban and rural older residents in practice? If the different outcomes of health in LTCI between urban and rural residents can be perceived at the primary stage, the chronic spread of this adverse result can be effectively prevented. The quasi-experimental examination of pilot cities in China, combined with data from the China Health and Retirement Longitudinal Study (CHARLS), provides an opportunity to explore the health outcome of LTCI and discrepancies between older urban and rural adults.

Thirdly, we identify the conclusion of this study will not only contribute to adjusting the LTCI policy in China but also deliver more implications for developing countries, especially those preparing to propel LTCI projects in the future. The intensified aging population has spilt over into all countries after the second world war^35^. Yet, it is worth noting that developing countries will become the chief battlefield of global population aging in the coming decades^36^. As the largest experimental plot of LTCI, policy evaluation in China will inevitably provide imperative practical references for other developing countries.

## Methods

### Data

The CHARLS was utilized in this study, which is similar to the Health and Retirement Survey (HRS) and the Survey of Health, Aging, and Retirement in Europe (SHARE). The CHARLS, a follow-up survey, aims to collect a set of high-quality microdata representing households and individuals who are aged 45 years and above in China. The database adopts multi-stage sampling, together with the Probability Proportionate to Size Sampling (PPS) method in the county/district and village sampling stages, and pioneers the electronic mapping soft technology (CHARLS-GIS) using the map method to construct the village-level sampling frame. The CHARLS questionnaire included basic personal information, family and family member information, health status, cognition and depression, health service utilization and medical insurance, income and so on. The Ethics Committee of Peking University approved this survey and we confirm all methods were performed in accordance with the relevant guidelines and regulations.

We have sorted out the specific implementation time of each pilot city according to the policy texts to better evaluate the effect of LTCI. Although the Ministry of Human Resources and Social Security wanted to initiate LTCI in 2016, the time node of initiation has been inconsistent across pilot cities. As evident from Table 1, Qingdao was the first city to execute LTCI well before the nationwide first pilot batch. Qingdao initiated LTCI in urban areas in July 2012. It then expanded the scope to rural areas in January 2015, enabling it to become a pioneer city that had accomplished full coverage of LTCI. The investigation of CHARLS in 2015 was conducted from July to September, which was later than the launch in rural areas of Qingdao. Therefore, we regarded the rural samples of Qingdao in CHARLS 2015 as the treatment group. We should have covered 15 pilot cities, but only 12 cities were included because of the data limitations. Based on the baseline survey in 2011, we matched the data of 2015 and 2018, and then generated a three-phase panel data. Due to the survey in 2011 excluding Shihezi, Changchun, and Nantong, the treatment group only covers Suzhou, Shanghai, Qingdao, Jingmen, Shangrao, Guangzhou, Chengdu, Chengde, Anqing, Ningbo, Tsitsihar and Chongqing for the following empirical analysis.

**Table 1:** Details on the execution of LTCI in pilot cities.

Pilot cities	Year of starting LTCI	Coverage
Qingdao	2012 and 2015	Urban and Rural residents
Nantong	2016	
Jingmen; Shanghai; Shihezi; Suzhou	2017	
Shangrao	2016	Urban employees
Chengdu; Guangzhou	2017	
Changchun	2017	Urban residents
Chengde	2016	Urban employees in some districts
Anqing; Ningbo; Tsitsihar; Chongqing	2017	

The empirical analysis comprises three components. First, we evaluated the health outcomes of LTCI and whether different impact on health outcomes existed between urban and rural residents. In particular, Suzhou, Shanghai, Qingdao, Jingmen, were regarded as the treatment group due to the coverage of LTCI involving both urban and rural areas in these cities. Afterward, we deleted the samples of the other eight pilot cities in Table 1. Second, we further verified the health effects of LTCI for residents in urban areas among pilot cities. Some pilot cities whose coverage only involves some districts were dropped. We regarded Suzhou, Shanghai, Shangrao, Qingdao, Jingmen, Guangzhou, and Chengdu as the samples of the treatment group in this part. Among them, four pilot cities, including Suzhou, Shanghai, Qingdao, Jingmen, achieved full coverage of urban and rural residents. Notably, the sample in this study includes only urban adults in order to explicitly investigate the impact of LTCI on residents in urban areas. Third, we analyzed the sensitivity of the outcomes in the second part.

### Variables and measurement

#### Dependent variable

The dependent variable of this study contains physical health and mental health. Physical health was measured by the individual’s self-rated health (SRH). Individuals were requested to report their current physical health status. The answer was a five-point Likert scale that 5–1 represented “very good”, “good”, “fair”, “poor”, and “very poor”, correspondingly. This question was a subjective health indicator, akin to Self-Rated Health measured by the Likert five-point scale, a scale widely applied in the field of public health and epidemiology^37,38^.

Additionally, depression scores were used to explore the effect of LTCI implementation on mental health among residents, and depression symptoms were used to measure mental health. The CHARLS measured depressive symptoms via the Center for Epidemiological Studies Depression Scale (CED-S). The scale included 10 questions, each with 4 possible responses: 1 for “almost none,” 2 for “sometimes,” 3 for “often,” and 4 for “most of the time.” After reverse coding the positive questions, we calculated 10 answers to these questions to obtain the final depression score. The higher the score, the worse the mental health status.

#### Independent variable

The interaction term between the treatment group and the dummy variable before and after implementation of LTCI was employed in this study.

#### Control variable

The inclusion of control variables is necessary for the DID model, and the control variables must be independent of the policy. Considering the characteristics of dependent variables, we predominantly focused on the individual-level factors including age, gender, marital status, the number of children alive, and socioeconomic variables involving the education level and per capita expenditure. All of these variables have an impact on the health of older adults, and the inclusion of these variables can improve the goodness of model’s fit. In addition, since the policy is at the municipal level and which city belongs to the treatment group may not be entirely exogenous, it is necessary to include variables at the municipal level. The differences of LTCI between cities, such as economic characteristics, medical resources, and other characteristics, may affect whether the city belongs to the treatment group. Therefore, we have added the variables of GDP, general public budget expansion, and the number of licensed (assistant) physicians.

The measurements of variables and basic characteristics were presented in Table 2. In this study, about 82% of the sample were rural residents, but only 18% were urban residents. The proportion of rural residents in the sample was relatively higher, which was determined by the original sampling method of the CHARLS. The SRH level of total residents was comparatively low, and only 24.75% and 21.29% of residents’ health were above the good level in urban and rural areas, respectively. The mean of depression symptoms of total residents was 9.6. The average age of total residents was 61.85. The average educational year of urban and rural residents were 8.54 and 4.72, respectively. 51.58% of the urban residents were male, compared with 46.47% of the rural residents. Additionally, 84.54% of residents had a spouse, and 69.37% of residents obtained care. The average number of children alive of total residents was 2.72.

**Table 2:** Descriptive statistics of variables.

Variable	Definition	Total residents	Urban residents	Rural residents
Self-rated health	Ordered variable:			
	5, very good;	9.12%	8.51%	9.26%
	4 good;	12.8%	16.24%	12.03%
	3 fair;	49%	54.57%	47.78%
	2 poor;	23.2%	17.26%	24.37%
	1 very poor	5.98%	3.41%	6.55%
Depression symptoms	Continuous variable: depression score, from 0 to 30 (mean)	9.6	7.24	10.12
Age	An individual’s age	61.85	63.2	61.5
Gender	Dummy variable:			
	1, male;	47.4%	51.58%	46.47%
	0 female	52.6%	48.42%	53.53%
Education Level	An individual’s educational year	5.41	8.54	4.72
Marital status	Dummy variable:			
	1, have a spouse;	84.54%	83.54%	84.76%
	0 have no spouse	15.46%	16.46%	15.24%
Hukou	Dummy variable:			
	1, Urban;	18.12%	–	–
	0 Rural	81.88%	–	–
Per capita expenditure (with logarithm)	Calculated according to the actual circumstance	7.58	8.13	7.45
Health status when young	Ordered variable:			
	1, very good;	10.73%	12.8%	10.27%
	2 good;	39.06%	41.35%	38.55%
	3 fair;	22.05%	22.43%	21.95%
	4 poor;	21.23%	17.72%	22.01%
	5 very poor	6.94%	5.7%	7.22%
Whether obtain care	Dummy variable:			
	1, Yes;	69.37%	67.89%	69.7%
	0 No	30.63%	32.11%	30.3%
Number of children alive	Calculated according to the actual circumstance	2.72	2.25	2.82
Gross domestic product in earch city (with logarithm)	Calculated according to the actual circumstance	16.5	–	–
General public budget expenditure in earch city (with logarithm)	Calculated according to the actual circumstance	14.75	–	–
Number of licensed (assistant) physicians in earch city (with logarithm)	Calculated according to the actual circumstance	8.98	–	–

Mean were performed for continuous variables. N (%) were performed for categorical variables.

### Model

We used a differences-in-differences (DID) specification to evaluate the health effects of LTCI. The benchmark model was set as follows:

1 $$Y_{itj} = \, \beta_{0} + \beta_{1} Treat_{ij} \times \, Post_{it} + \, \beta_{2} Z_{ijt} + \, \alpha_{i} + \, U_{t} + \, \xi_{ijt}$$

In Eq. (1), *Y*_*itj*_ represents a set of individual-level dependent variables, chiefly referring to SRH and depression scores. *Treat*_*ij*_ × *Post*_*it*_ denotes the interaction term between the treatment group and the dummy variable before and after the implementation of LTCI. Moreover, *β*_*1*_ is the coefficient of the interaction term representing the difference in outcome variables between residents in cities with and without LTCI, which determined whether the effect of LTCI improved physical and mental health. *Z*_*ijt*_ specifies a set of individual-level covariates, including age, gender, marital status, education level, and household expenditure, and *α*_*i*_*, U*_*t*,_ and *ξ*_*ijt*_ represent the year fixed effect, individual fixed effect, and the random error term, respectively. Almost all of the statistical analyses were performed with Stata 16.0 and robust standard error was applied in all models.

### Ethics approval and consent to participate

The data used in this study were retrieved from the China Health and Retirement Longitudinal Study (CHARLS). This survey was endorsed by the Biomedical Ethics Committee of Peking University (NO. IRB00001052–11015). All participants in the survey signed or marked (if illiterate) the informed consent forms.

## Results

### Health effects of LTCI

As depicted in column (1), (2), and (3) of Table 3, the implementation of LTCI has effectively improved the SRH of the whole group of residents; however, this effect may only be significant for the group of urban residents. Hence, this finding may illustrate the differences in health outcomes of LTCI between urban and rural residents. We assessed the relationship between SRH and mental health among urban residents to further evaluate the impact of LTCI on urban residents. The implementation of LTCI might have a positive effect on the SRH of urban residents but not on their self-rated depression scores (refer to column (4) and (5) in Table 3). Specifically, the execution of LTCI can improve the SRH of urban residents by 0.377 units in comparison to the urban residents without LTCI.

**Table 3:** Health effects of LTCI on older residents.

SRH	(1) Total residents	(2) Urban residents	(3) Rural residents	(4) SRH (urban residents)	(5) Self-rated depression scores (urban residents)
Heterogenous Timing DID	0.357*** (0.113)	0.605*** (0.135)	0.222 (0.15)	0.377*** (0.144)	− 0.411 (0.677)
Control variable	YES	YES	YES	YES	YES
N	12,506	2335	10,168	2364	2264

*p < 0.10, **p < 0.05, ***p < 0.01.

### Parallel trend test

The assumption of parallel trend is the premise for using DID in empirical research because DID can only be employed when the dependent variables in the treatment and control groups conform to the assumption of parallel trend before the policy implementation. Parallel trend test aims to examine whether the interaction term is significant before the policy implementation. If the result is not significant, DID could be utilized. Despite the staggered DID method having been applied in this study; it is impossible to conduct staggered DID parallel trend test because the data is only three phases. Hence, we adopt the following measures to overcome the shortcoming.

**Figure 1:**
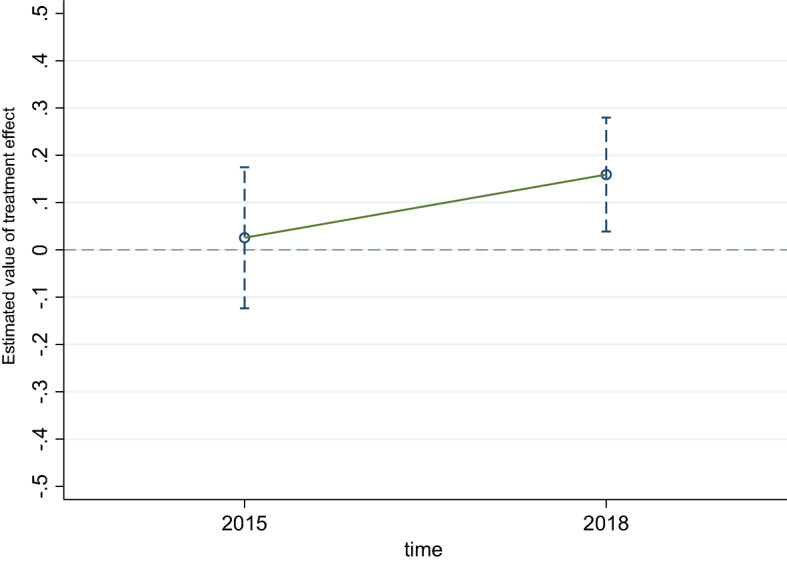
Parallel trend test—total residents.

**Figure 2:**
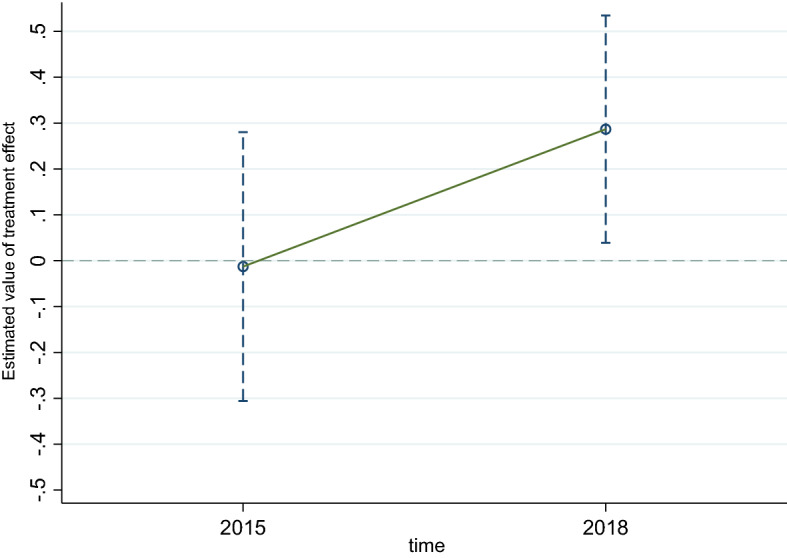
Parallel trend test—urban residents.

First, we excluded Qingdao, which has implemented LTCI before 2016. Second, we utilized the data from the year 2011, 2015, and 2018 to conduct parallel trend test. Meanwhile, we regarded the year 2011 and 2015 as before the policy implementation, and year 2018 as after the policy implementation. Third, we adopt event analysis to obtain the dynamic effect of LTCI on the residents’ health. Figures 1 and 2 illustrate the dynamic effects of LTCI on the health outcomes of all residents and only urban residents, respectively. The sample range of all residents and urban residents is detailed in below Table 1. According to Figs. 1 and 2, whether the analysis object is all older residents or only for the urban group, the estimated value of the coefficient is not significant before LTCI is implemented, that is, the treatment and control group meet the parallel trend assumption.

### Placebo effect test

In the placebo effect test, falsification tests were mainly adopted to verify the plausibility of the LTCI effect. The placebo effect test has a critical role in the model estimation, which could improve the robustness of the estimation results. The core idea is to fictionalize the processing team or estimate the time for fictitious policies. We first fabricated another treatment group, used rural samples, and regarded pilot cities without covering rural areas as treatment groups, including Chengde, Ningbo, Anqing, Guangzhou, Chengdu, Chongqing, Shangrao, and Tsitsihar. Notably, the control group constitutes other non-pilot cities (i.e., deleting the samples of the other four pilot cities whose coverage includes both rural and urban areas). The findings were not significant as shown in Table 4, which may prove that the implementation of LTCI can improve the residents’ health effectively.

**Table 4:** Sensitivity analysis of LTCI implementation.

SRH	Dummy treatment group	Dummy time of implementing LTCI	Health status when young
DID	0.01 (0.112)	0.2 (0.217)	0.31 (0.225)
Control variable	YES	YES	YES
N	10,513	1293	2364

*p < 0.10, **p < 0.05, ***p < 0.01.

Second, we employed the method of fabricating policy implementation time and regarded the years before LTCI implementation as dummy policy implementation time. We deleted the rural data in the sample and retained the urban sample for this analysis. As complete coverage of the urban area in Qingdao has been achieved since 2012, this sample was deleted. The pilot cities that only implemented LTCI after 2016 were retained in the final sample, including Suzhou, Shanghai, Shangrao, Jingmen, Guangzhou, and Chengdu. Moreover, Chengde, Anqing, Ningbo, Tsitsihar, and Chongqing were deleted as they only involved some districts. In this section, only data from 2011 and 2015 were used. We fictitiously set the policy implementation time as 2015; hence, 2011 is before the policy, and 2015 is after the policy. If the regression result of the DID estimator in this analysis is still significant, it suggests that the original estimation result may be biased. Further, the result of Model 2 in Table 4 is not significant, which to some extent verifies that the improvement in health levels is indeed caused by the implementation of LTCI.

Finally, we chose a factor unaffected by policy intervention as the dependent variable for regression analysis. The residents’ health at a young age was selected as the dependent variable. Health at a young age occurs before LTCI policy; thus, health at a young age is completely unaffected by LTCI. In this part, the rural data in the sample were deleted and only the urban sample was retained. The pilot cities, including Chengde, Anqing, Ningbo, Tsitsihar, and Chongqing, that only involved some districts were deleted. Moreover, we sorted Suzhou, Shanghai, Shangrao, Qingdao, Jingmen, Guangzhou, and Chengdu into the treatment group. The result of model (3) in Table 4 is not significant, which verifies that the LTCI could improve the health of residents to some extent.

## Discussion

In this study, the three-period data from CHARLS in 2011, 2015, and 2018 were utilized to evaluate the health effects of LTCI on older residents within the triple difference framework. The empirical results demonstrated that the implementation of LTCI in China could effectively improve the SRH of older residents, which is consistent with the conclusion of a previous study^39^. Further analysis after separating the urban and rural samples illustrated that LTCI could only improve the SRH of urban older residents effectively and had no impact on older rural residents. Based on the aforementioned results, we inferred that LTCI may induce diverse impact on health outcomes among older residents in urban and rural areas to a certain degree. Despite basic medical insurance being an incontrovertible role in older adults’ health, disparate impact on health outcomes provoked by LTCI between urban and rural older residents should be addressed as well. This difference might be related to the considerable disparity in access to services for LTCI residents in China^40^. For most older adults who require long-term care, the cost is the greatest burden. As many older people have never had a formal job, they do not have a pension and cannot afford out-of-pocket expenses^27^. This circumstance is more widespread in rural China. Hence, we propose to provide more financial support to the low-income older population in order to promote their preference for LTCI^41^.

LTCI may have exclusiveness to older rural people due to some restrictions on the entry threshold. Most pilot cities in China primarily relied on UEBMI funds to finance the LTCI^42^. This dependence produces a lack of equal access to long-term care services for rural residents without UEBMI. However, existing studies have estimated that aging would double the LTC expenses by 2030^32^. As fears grow about the burden of government finance, the establishment of a reasonable individual-social-government payment mechanism might be essential^43^. The relative deprivation may have a greater negative impact on older rural people, which could explain the finding that poorer health status was associated with low income^44^. Moreover, low income further resulted in older rural adults acquiring less individual social capital, thus affecting their health-related quality of life^45^. Personal characteristics, such as income and education level, may damage SRH and drive health inequalities among older adults^46,47^. Our results revealed that this different impact was involved in LTCI and the health of older rural residents cannot be remarkably improved.

Apart from individual characteristics, the disadvantages of the rural healthcare delivery system in China introduced challenges to health security among older adults as well^28^. The Chinese LTCI was launched relatively late, and there are only a handful of pilot cities to date. In most middle-income countries, it is impractical to provide enough infrastructure for long-term care services due to the unprecedented acceleration of aging^48^. Nonetheless, the scarce infrastructure services for LTCI are more severe in China, especially in rural regions. The long-term care workforce shortage and weak quality assurance are concerning^49^. In particular, the outbreak of COVID-19 has restricted population mobility, it is worth reconsidering how to achieve the goal of LTC adequately on the occasion of no labor force to care for the elderly. Previous scholars have realized that older adults suffered from inequities in healthcare, including little or no access to facilities, trained personnel, and drugs^50^. Unfortunately, the different impact on health accompanying LTCI are even more detrimental in rural China, which was omitted in the existing literature.

Therefore, we believe that the policymakers of LTCI should give more attention to the rural older group who are in great need of long-term care. Policymakers have to extend long-term care coverage to rural areas to improve the quality of life for older adults^51^. Despite being in a different social background from middle-income countries, the shortages of facilities and funding for long-term care are equally grim in China, which governments need to focus on^52^. A combination of formal and informal care might be one of the alternatives to meet urgent long-term care needs^53^. Still, the development of long-term care for families and communities is correspondingly sluggish in China^49^. It is necessary for China to implement overall LTCI to cope with the issues related to aging as soon as possible. Although the Chinese long-term care system is characterized by increasing involvement of the private sector^49^, there is a particular need to develop public LTCI as a solid foundation^54^. LTCI is intended to provide more financial support to low-income groups^24^.

## Conclusions

Despite the implementation of LTCI improving the residents’ health, this result may only be effective for urban groups. In China, LTCI may have enticed different impact on health outcomes between urban and rural areas, which might be ignored by scholars and policymakers more or less. Therefore, governments and policymakers must focus on older rural adults as they are in the greatest need of long-term care.

However, there are several limitations of this study. Firstly, due to the original sampling method of CHARLS, the proportion of urban residents is much higher than rural residents. The huge disparity in sample size may interfere with the results to some extent, nevertheless, CHARLS is currently the most suitable and accessible database to evaluate the effect of LTCI in China. Secondly, the measurement of health indicators is derived from SRH. This indicator can represent the health level of residents, but it may be subjective. Scholars can consider using more accurate and objective health indicators for further investigation. Thirdly, the statistical scale of CHARLS might be unable to scrupulously verify the influencing mechanism between LTCI and residents’ health. Apart from LTCI, we failed to consider other policies having an impact on the health of older adults.

## Data Availability

The datasets used during the current study are available from the corresponding author on reasonable request.

## Abbreviations

LTC: Long-term care
LTCI: Long-term care insurance
DID: Difference-in-differences
CHARLS: China Health and Retirement Longitudinal Study
UEBMI: Urban employees’ basic medical insurance
URBMI: Urban resident basic medical insurance
COVID-19: Coronavirus disease 2019
